# Comparing multiple definitions of obesity in a large nationwide health program

**DOI:** 10.1038/s41598-026-53073-7

**Published:** 2026-05-26

**Authors:** Gergő József Szőllősi, Orsolya Csenteri, Péter Andréka, Lilla Andréka, Zoltán Jancsó, Péter Vajer

**Affiliations:** 1 Gottsegen National Cardiovascular Center, Haller u. 29, Budapest, 1096 Hungary; 2 Coordination Center for Research in Social Sciences, Faculty of Economics and Business, University of Debrecen, Debrecen, Hungary; 3 Doctoral College of Semmelweis University, Budapest, 1085 Hungary; 4 Department of Family Medicine, Semmelweis University, Budapest, 1085 Hungary

**Keywords:** Obesity, Epidemiology, Body mass index, Diseases, Endocrinology, Health care, Medical research, Risk factors

## Abstract

Obesity represents a major global health challenge, yet its prevalence and clinical significance may vary substantially depending on the definition applied. While the Body Mass Index (BMI) remains the most widely used indicator, alternative frameworks such as the European Association for the Study of Obesity (EASO) classification, the metabolically healthy obesity (MHO) concept, and the recent Lancet functional framework offer broader, clinically oriented perspectives. We conducted a cross-sectional, population-based analysis of 108,350 adults. BMI was calculated from measured height and weight, with overweight defined as 25.0–29.9 kg/m² and obesity as ≥ 30.0 kg/m². EASO obesity classification incorporated both BMI and the presence of obesity-related complications. Metabolic health was assessed using established cut-offs for hypertension, diabetes, and dyslipidaemia, and MHO was defined as BMI ≥ 30.0 kg/m² without metabolic abnormalities. By conventional BMI cut-offs 77,393 participants were overweight or obese, within which 47.2% were obese and 52.8% were overweight, respectively. In contrast, the EASO classification identified 29,044 respondents of the total population as obese in the overweight strata, leaving only 11,815 as non-obese. Therefore, among individuals with BMI 25.0–29.9, 71.1% were classified as obese by EASO criteria. Among participants with BMI-defined obesity, 12,151 (35.0%) were metabolically healthy obese and 22,585 (65.0%) were considered metabolically unhealthy. In terms of comorbidities, 82.8% (*n* = 24,521) of obese were clinically obese and 17.2% (*n* = 5,094) were pre-clinical obese. Obesity prevalence and the identification of metabolically healthy subgroups depend on the definition applied. While BMI alone provides a conservative estimate, other frameworks capture a much broader population. These findings highlight the need for unified definitions that may better reflect clinical risk and guide both public health surveillance and individual patient management.

## Introduction

Obesity remains one of the most pressing public health challenges worldwide, contributing to the global burden of cardiovascular disease, type 2 diabetes, cancer, and premature mortality. This is further aggravated by the fact that prevalence rates are particularly high in Central and Eastern Europe, including Hungary, where obesity contributes substantially to morbidity and healthcare expenditure^1–3^.

Body Mass Index (BMI) is the most widely used and easily measurable indicator for assessing overweight (≥ 25.0 kg/m²) and obesity (≥ 30.0 kg/m²). However, BMI has major limitations, since it does not distinguish between fat mass and lean mass, fails to capture body fat distribution, and provides no information about metabolic health or functional impairment. The clinical relevance of these limitations lies in the fact that central or visceral adiposity is more strongly associated with cardiometabolic risk than peripheral fat accumulation. Consequently, individuals with the same BMI may have different cardiometabolic risk profiles depending on adiposity distribution^4–8^.

To overcome these limitations, new conceptual frameworks have emerged that redefine obesity as a disease of adiposity-associated dysfunction rather than excess weight alone. The European Association for the Study of Obesity (EASO) has long advocated a clinical approach that integrates anthropometric, metabolic, and functional parameters^9^. More recently, the 2025 Lancet Diabetes & Endocrinology Commission on the Definition and Diagnosis of Obesity as a Disease proposed a functional classification framework, distinguishing between preclinical obesity (presence of risk without overt complications) and clinical obesity (manifest obesity-related health impairment). This approach reframes obesity as a progressive, multidimensional disease requiring stratified management rather than a binary weight status^10^. Parallel to these developments, the concept of metabolically healthy obesity (MHO) has gained attention, referring to individuals with obesity who lack major metabolic abnormalities such as hypertension, dyslipidaemia, or diabetes^11^. Although debated, this phenotype highlights the heterogeneity of obesity and the possible need for multidimensional assessment^12–15^.

Because of the multiple definitions that differ across frameworks, prevalence estimates and clinical interpretations of obesity may vary widely, hence complicating surveillance, treatment eligibility, and resource allocation. Therefore, multiple diagnostic systems, such as BMI thresholds, EASO categories, metabolic health criteria, and the new functional staging framework coexist in research and practice, each emphasizing different dimensions of disease. For clinicians, this diversity can be confusing, as it is not always clear which definition best guides patient management or risk stratification. Nevertheless, these parallel approaches each serve a specific purpose, while BMI offers simplicity and comparability, functional and metabolic classifications capture clinical relevance and disease progression.

## Methods

To address the current ambiguity created by parallel diagnostic systems, this study compares how complementary obesity definitions operate in practice. Specifically, we examine obesity prevalence and classification across BMI-based thresholds, the EASO clinical framework, and the functional (Lancet Commission) framework, alongside metabolic-health definitions, to provide a population-level characterization of obesity heterogeneity and its clinical implications. Therefore, the main objective was to compare the prevalence of obesity based on different definitions in a large national sample.

This cross-sectional, population-based analysis was conducted using a large nationwide dataset from adults aged 40–65 years in Hungary (initial *n* = 109,889), collected within the frameworks of “Three Generations for Health” program. This program was a national prevention program established by Government Decision No. 1234/2017 (IV. 28.), with the aim of strengthening cardiovascular prevention mainly in primary care in Hungary. The program was implemented through participating general practices with professional and IT support provided by the Gottsegen National Cardiovascular Center. Data were collected within the operational framework of the program through participating primary care providers and related activities, using standardized data recording procedures. Participation depended on enrolment in participating practices and consent to program-related assessment. Hence, participating adults underwent anthropometric measurements, clinical assessment, and laboratory testing according to standardized procedures. Data were anonymized before analysis. After excluding records with missing BMI values (*n* = 1,539), the final analytic sample comprised 108,350 participants. All anthropometric and laboratory data were collected following standardized procedures. Waist circumference (cm) and waist-to-height ratio were also recorded to support central obesity assessment. Height and weight were directly measured, and Body Mass Index (BMI) was calculated as weight in kilograms divided by height in meters squared (kg/m²). First, overweight was defined as BMI 25.0–29.9 kg/m² and obesity as BMI ≥ 30.0 kg/m² according to World Health Organization criteria^3^. Second, the EASO clinical framework was approximated by combining anthropometry with cardiometabolic complications: individuals with obesity-related conditions (e.g., hypertension, dyslipidemia, or type 2 diabetes) were considered EASO-obese, whereas those without such complications were classified as EASO non-obese (overweight individuals with ≥ 1 complication were also treated as EASO-obese to reflect the complication-anchored nature of this scheme). Third, the functional (Lancet Commission) classification was applied to participants with BMI ≥ 30.0 kg/m² to distinguish pre-clinical obesity, highlighting elevated adiposity with early risk but with no manifest complication, from clinical obesity, defined by the presence of obesity-related health impairment(s), comorbidity, or functional limitation; this distinction was operationalized from the available cardiometabolic diagnoses and treatment information. Fourth, metabolic health status was derived to identify metabolically healthy obesity (MHO) versus metabolically unhealthy obesity (MUO): MHO required BMI ≥ 30.0 kg/m² and the absence of major abnormalities (elevated blood pressure ≥ 130/85 mmHg or antihypertensive therapy, triglycerides ≥ 1.7 mmol/L or lipid-lowering therapy, HDL < 1.0 mmol/L in men or < 1.3 mmol/L in women, fasting glucose ≥ 5.6 mmol/L or diagnosed type 2 diabetes, or use of relevant medications). Additional covariates included waist circumference, lipid parameters, blood pressure, fasting glucose, and self-reported or medically confirmed treatment for hypertension, dyslipidemia, or diabetes. Central obesity was defined as waist circumference ≥ 94 cm for men and ≥ 80 cm for women.

To compare how different obesity definitions classify individuals within the same dataset, these four classification systems were operationalized using harmonized procedures and the same underlying measurements. These included BMI-based WHO criteria, an EASO-informed classification, a functional staging approximation based on the Lancet framework, and metabolic-health classification. The criteria and their operationalization in the present study are summarized in Table 1.

**Table 1:** Classification frameworks and operational criteria used to define obesity phenotypes in the present study.

Classification framework	Core concept	Operational criteria used in this study	Interpretation
**BMI-based WHO classification** ^3^	Obesity defined by body size using BMI thresholds	Overweight: BMI 25.0–29.9 kg/m²; obesity: BMI ≥ 30.0 kg/m²	Simple anthropometric classification; does not incorporate metabolic status, complications, or functional impairment
**EASO-informed classification** ^9^	Obesity conceptualized as excess adiposity associated with health impairment or complications	Individuals with BMI ≥ 30.0 kg/m² were classified as obese. Individuals with BMI 25.0–29.9 kg/m² were also classified as obese if they had at least one obesity-related complication, including hypertension, dyslipidemia, or type 2 diabetes	Captures individuals below the classical BMI obesity threshold who already show obesity-related clinical risk
**The Lancet functional framework** ^10^	Obesity classified according to the presence or absence of obesity-related clinical manifestations or functional impairment	Applied among individuals with BMI ≥ 30.0 kg/m². Clinical obesity was approximated by the presence of available obesity-related health impairment indicators or comorbidities in the dataset. Preclinical obesity was defined as BMI ≥ 30.0 kg/m² without these available indicators	Represents a pragmatic approximation of the functional framework using available cardiometabolic and treatment data; direct measures of functional limitation were not available
**Metabolic health classification** ^11^	Obesity phenotyped according to the presence or absence of metabolic abnormalities	MHO: BMI ≥ 30.0 kg/m² and absence of major metabolic abnormalities: elevated blood pressure or antihypertensive treatment, triglycerides ≥ 1.7 mmol/L or lipid-lowering therapy, low HDL cholesterol, elevated fasting glucose or type 2 diabetes, or relevant medication use. MUO: BMI ≥ 30 kg/m² with at least one of these abnormalities	Distinguishes metabolically healthy and metabolically unhealthy obesity phenotypes among individuals with BMI-defined obesity

Analyses were conducted using Stata 13. Descriptive statistics (frequencies and percentages) were calculated for each classification system. Cross-tabulations were used to examine overlap and concordance between BMI-based, EASO, metabolic, and functional definitions. Missing data were reported explicitly, and proportions were calculated both within each framework and against the total cohort. Group differences were tested using Pearson’s chi-square test where appropriate; however, the analysis primarily focused on prevalence estimates and descriptive comparisons rather than inferential statistics.

## Results

**Fig. 1:**
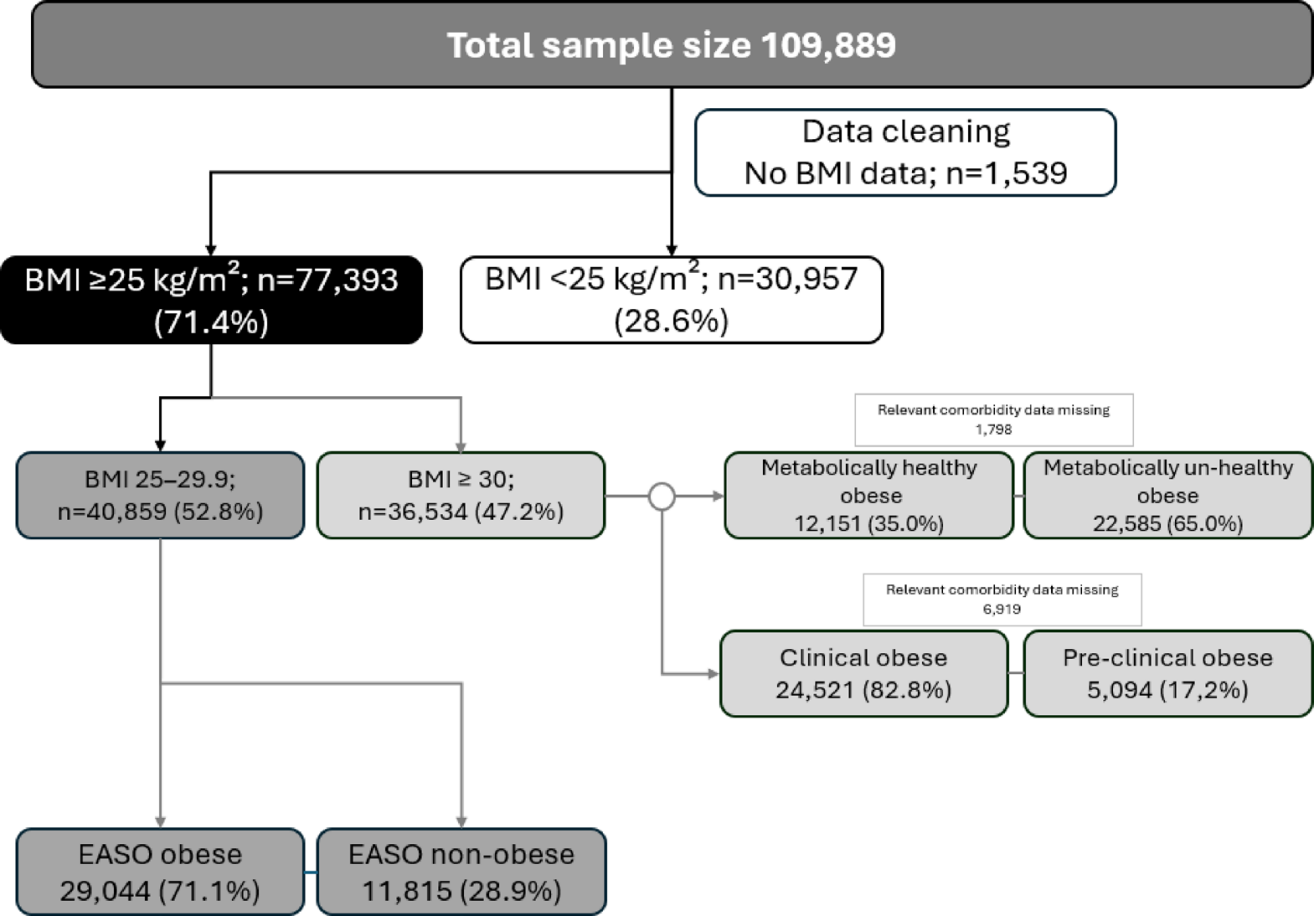
Cohort flow and parallel classification of participants by BMI thresholds, EASO criteria, metabolic health status (MHO/MUO), and functional (clinical vs. pre-clinical) obesity. Note: Metabolic-health status and functional stage are shown as parallel classification pathways within the BMI ≥ 30 kg/m² group, reflecting that these frameworks describe distinct but complementary dimensions of obesity.

The initial cohort comprised 109,889 adults (40–65 years). BMI data were unavailable for 1,539 participants (1.4%), leaving 108,350 with BMI data as demonstrated in Fig. 1. Among those with BMI data, 77,393 (71.4%) had BMI ≥ 25.0 kg/m² and 30,957 (28.6%) had BMI < 25.0 kg/m². Within the BMI ≥ 25.0 group, 40,859 (52.8%) were overweight (25.0–29.9 kg/m²) and 36,534 (47.2%) had BMI ≥ 30.0 kg/m². Expressed against the full cohort, this corresponds to 37.2% overweight and 33.3% BMI ≥ 30.0, respectively.

Applying the EASO framework within the overweight stratum (*n* = 40,859) identified 29,044 (71.1%) as EASO-obese and 11,815 (28.9%) as EASO-non-obese.

For metabolic-health phenotyping in the BMI ≥ 30.0 group (*n* = 36,534), comorbidity data were available for 34,736 (95.1%); 1,798 (4.9%) were missing. Among those with complete data, 12,151 (35.0%) were metabolically healthy obese (MHO) and 22,585 (65.0%) were metabolically unhealthy obese (MUO). When referenced to the entire BMI ≥ 30.0 group (including missing), MHO and MUO represented 33.3% and 61.8%, respectively, with 4.9% unclassifiable due to missing relevant comorbidity information. Relative to the total cohort, MHO and MUO accounted for 11.2% and 20.9%, respectively.

Clinical staging could be ascertained for 29,615 (81.1%) of those with BMI ≥ 30.0; data were missing for 6,919 (18.9%). Among evaluable individuals, 24,521 (82.8%) were clinically obese and 5,094 (17.2%) were pre-clinical obese. Against the full BMI ≥ 30.0 group (including missing), these correspond to 67.1% clinical obesity, 13.9% pre-clinical obesity, and 18.9% unclassified. As proportions of the total cohort, clinically obese and pre-clinical obese represented 22.6% and 4.7%, respectively.

Within the subset where both clinical staging and metabolic health information were available (*n* = 28,471), a clear association (*p* < 0.001) was observed between metabolic status and clinical severity of obesity (Table 2). Among those classified as clinically obese (*n* = 23,834), 76.2% (*n* = 18,172) were metabolically unhealthy, while 23.8% (*n* = 5,662) met the criteria for metabolically healthy obesity. In contrast, among pre-clinical obese individuals (*n* = 4,637), the vast majority (86.0%; *n* = 3,986) were metabolically healthy, and only 14.0% (*n* = 651) were metabolically unhealthy. Overall, across this subset, two-thirds (66.1%) were metabolically unhealthy and one-third (33.9%) metabolically healthy.

**Table 2:** Association between functional obesity stage and metabolic health status among individuals with BMI ≥ 30 kg/m².

Functional obesity stage	Metabolically healthy obese *n* (%)	Metabolically unhealthy obese *n* (%)	Total
**Clinical obesity**	5,662 (23.8)	18,172 (76.2)	23,834
**Preclinical obesity**	3,986 (86.0)	651 (14.0)	4,637
**Total**	9,648 (33.9)	18,823 (66.1)	28,471

Note: Percentages are row percentages. Only participants with complete data for both functional obesity staging and metabolic health classification were included.

Pearson’s chi-square test: *p* < 0.001.

## Discussion

In this large, population-based sample of 108,350 adults aged 40–65 years, the prevalence and distribution of obesity varied substantially depending on the applied definition. Using the conventional BMI-based approach, one-third of the population met criteria for obesity. Furthermore, the EASO framework identified an additional 29,044 individuals as obese beyond the 36,534 classified as such by the traditional BMI criteria, reclassifying approximately 71.4% of overweight individuals as obese due to the presence of obesity-related complications.

Within the obese (BMI ≥ 30.0) group, approximately one-third were classified as metabolically healthy obese, whereas two-thirds were metabolically unhealthy obese. Among individuals with BMI ≥ 30.0 kg/m² and available staging data, 82.8% were classified as clinically obese and 17.2% as pre-clinical obese according to the staging system published in The Lancet^10^. Thus, a strong association was observed between metabolic status and clinical severity of obesity, because most clinically obese individuals were metabolically unhealthy, while pre-clinical obesity was predominantly associated with metabolic health, which is consistent with the previous literature^11,16,17^.

Although MHO and preclinical obesity may overlap in real-world practice, they are not synonymous, since MHO is considered as a metabolic phenotype defined by the absence of major metabolic abnormalities, whereas preclinical obesity is considered as a staging concept referring to excess adiposity without evident obesity-related clinical manifestations or functional impairment. Therefore, neither category should be interpreted as indicating absence of risk or as a reason to limit preventive care. Rather, their clinical value lies in supporting more differentiated risk stratification, follow-up, and communication in primary care, while maintaining preventive intervention for all individuals with excess adiposity.

These findings also highlight the limitations of relying on BMI alone. Although the BMI-based definition provides a simple and reproducible measure, it may underestimate the true burden of obesity-related risk^18,19^. Many individuals with BMI between 25.0 and 29.9 kg/m² already exhibit comorbidities consistent with metabolic or clinical obesity, highlighting that BMI alone fails to distinguish between metabolically benign and harmful phenotypes. Nevertheless, metabolically benign obesity remains contested, since metabolically healthy obesity frequently transitions to an adverse metabolic profile over time, with higher cardiometabolic risk than normal-weight comparators^20–22^. Therefore, clarifying these phenotypes, including their prognostic value, might warrant further longitudinal research. Hence, cardiometabolic risk may contribute to more severe adverse health status even below standard BMI thresholds, supporting the need for earlier care. Given the divergence between BMI-based and metabolic definitions, exclusive use of BMI is prone to under-triage patients who would benefit from preventive care^23–26^.

For practicing family physicians, these definitional inconsistencies translate into practical uncertainty, which means that the same patient may or may not meet a threshold for obesity. That is why EASO staging and metabolic-health classification offer complementary information, the former focuses on functional/clinical impairment, while the latter highlights biological resilience or vulnerability. Aligning primary-care pathways with multidimensional criteria consistent with EASO and the Lancet functional framework could better match labels to need and support more targeted resource allocation^27^. Beyond clinical decision-making, these differences may also affect healthcare financing, treatment eligibility, and reimbursement. In systems where access to structured lifestyle programs, specialist referral, anti-obesity medications, or metabolic interventions depends on diagnostic thresholds, the chosen definition may determine which patients are recognized as eligible for care. BMI-only criteria may under-identify individuals with clinically relevant cardiometabolic risk, whereas broader complication-anchored or functional definitions may increase the population considered for intervention. This highlights the need for transparent and harmonized criteria that balance early risk identification with equitable and sustainable resource allocation.

Since traditional BMI thresholds do not capture the full spectrum of obesity-related disease burden, a pragmatic, multidimensional approach that integrates BMI with clinical and metabolic markers may help reduce diagnostic ambiguity, contributes to risk stratification and improve the timeliness of preventive care.

## Conclusion

This large, population-based analysis demonstrates that obesity is not a homogeneous disease but a multidimensional condition which prevalence and clinical significance depend strongly on the definition applied. Thereby, conventional BMI thresholds may underestimate the true burden of obesity-related disease, while clinical and functional classifications provide a more accurate reflection of health impairment. Thus, integrating anthropometric, metabolic, and functional criteria offers a more meaningful representation of obesity as a chronic, progressive disease rather than a simple measure of excess weight. Hence, establishing consensus on clinically relevant definitions is essential to harmonize research, improve patient stratification, and guide more effective prevention and treatment strategies at both clinical and population levels.

## Strengths and Limitations

Major strengths of this study include its large, population-based sample and the comprehensive, parallel evaluation of multiple obesity definitions, such as BMI-based, functional, and metabolic-health frameworks within the same cohort. This multidimensional approach provides a unique opportunity to assess how different classification systems overlap and diverge in identifying obesity at the population level.

However, several limitations should be acknowledged. The dataset was not a probability-based nationally representative survey, and the findings should therefore be interpreted in relation to adults aged 40–65 years reached through this national prevention program in Hungary. Furthermore, the cross-sectional design precludes causal inference, and direct measurements of body composition or visceral adiposity were not available. Operationalization of the frameworks relied on secondary variables derived from existing data rather than clinically adjudicated diagnoses, which may have introduced some degree of misclassification. In addition, the use of predefined international cut-offs may not fully account for population- or ethnicity-specific variability. Finally, a small proportion of missing data for comorbidity indicators could have led to minor underestimation of certain subgroups.

## Data Availability

The data underlying this article are not publicly available. Access may be requested by the corresponding author upon reasonable request.

## Abbreviations

BMI: Body Mass Index
EASO: European Association for the Study of Obesity
HDL: High—Density Lipoprotein
MHO: Metabolically Healthy Obesity
MUO: Metabolically Unhealthy Obesity
WHO: World Health Organization
